# Identification of transcription factors related to diabetic tubulointerstitial injury

**DOI:** 10.1186/s12967-023-04069-8

**Published:** 2023-03-29

**Authors:** Jialu Liu, Guangzhong Duan, Wenxia Yang, Shumin Zhang, Fuyou Liu, Youming Peng, Lin Sun, Yu Liu, Li Xiao

**Affiliations:** 1 Department of Nephrology, The Second Xiangya Hospital, Central South University, Changsha, 410011 Hunan China; 2 Hunan Communication Polytechnic, Changsha, 410132 Hunan China

**Keywords:** Diabetic nephropathies, Tubulointerstitial injury, Transcription factor, Computational biology, Regulatory network

## Abstract

**Background:**

Diabetic nephropathy (DN) is a main cause of chronic renal failure. Despite decades of extensive study, the molecular mechanisms underlying diabetic tubulointerstitial injury remain unclear. We aim to identify key transcription factor genes involved in diabetic tubulointerstitial injury.

**Methods:**

A microarray dataset (GSE30122) from Gene Expression Omnibus (GEO) was downloaded. A total of 38 transcription factor genes based on 166 differentially expressed genes (DEGs) were identified by UCSC_TFBS.

**Results:**

The regulatory network showed connections between the top 10 transcription factors and their target DEGs. Gene Ontology (GO) enrichment and Kyoto Encyclopedia of Genes and Genomes (KEGG) pathway analysis of targeted DEGs indicated that extracellular space, extracellular exosome, cell surface and complement and coagulation cascades were most significantly enriched. Utilizing Nephroseq v5 online platform, the mRNA expression pattern analysis of transcription factor genes demonstrated that mRNA expression of *CDC5*, *CEBPA*, *FAC1*, *HFH1*, *IRF1*, *NFE2* and *TGIF1* increased in renal tubulointerstitium of DN patients compared with normal controls while that of *CEBPB* and *FOXO4* decreased in renal tubulointerstitium of DN patients compared with normal controls. Correlation analysis between mRNA expression of transcription factor genes in renal tubulointerstitium and clinical features showed that *AP1*, *BACH1*, *CDC5*, *FAC1*, *FOXD1*, *FOXJ2*, *FOXO1*, *FOXO4*, *HFH1*, *IRF1*, *POU3F2*, *SOX5*, *SOX9*, *RSRFC4*, *S8* and *TGIF1* may be related to diabetic tubulointerstitial injury.

**Conclusions:**

(1) *CDC5*, *FAC1*, FOXO4, *HFH1*, *IRF1* and *TGIF1* may be key transcription factor genes. (2)Transcription factors involved in diabetic tubulointerstitial injury may become prospective targets for diagnosis and treatment of DN.

**Supplementary Information:**

The online version contains supplementary material available at 10.1186/s12967-023-04069-8.

## Background

As a common complication of diabetes, diabetic nephropathy (DN) has become a primary cause of end stage renal disease (ESRD) worldwide [1, 2]. It was demonstrated that glomerular lesions play a key role in the early occurrence and development of DN. However, studies in recent years have also shown that tubulointerstitial changes contribute to development and progression of DN, even independently of glomerular lesions [3, 4]. Besides, proximal tubulopathy has been viewed as a key initial factor in the progression of DN [5]. Certain transcription factors (TFs) have been demonstrated to play an important role in these pathologies [6]. Clinical data has showed positive correlations between the transcription factors in tubular epithelial cells and DN progression, such as zinc-finger transcription factor snail homolog 1(Snai1) [7], X-box binding protein 1(XBP1) [8], hypoxia-inducible factor-1α(HIF-1α) [9], and nuclear factor of activated T cells 1(NFATc1) [10]. With in vivo studies, myocardin-related transcription factor A (MRTF-A) can promote transcription of type I and II collagen in an epigenetic manner [11]. Hypoxia-inducible factor (HIF-1) has been reported to mediate renal tubulointerstitial fibrosis and tubular injury in a murine model of type 1 diabetes [12, 13]. Yin Yang 1 (YY1) has been shown to accelerate renal fibrosis in db/db mice by upregulating α-SMA expression and epithelial-mesenchymal transition (EMT) [14]. In addition, our previous studies have also demonstrated that Rap1b ameliorates diabetic tubular injury [15], and the inhibition of NFATc1/TRPC6 signaling mitigates diabetic tubulointerstitial inflammation with in vivo and in vitro study [10]. Despite decades of extensive study, the molecular mechanisms underlying diabetic tubulointerstitial injury remain unclear. Thus, it is of great significance to identify key TFs associated with diabetic tubulointerstitial injury, as specific therapeutics can then be developed to target activation of selected TFs.

Recently, bioinformatic methods have been broadly employed to screen differentially expressed genes (DEGs) and transcription factor genes. As research reported, the differentially expressed genes (DEGs) were screened out and functional annotation was performed to identify TFs that regulate these DEGs. In this study, a mRNA microarray dataset downloaded from Gene Expression Omnibus (GEO) was used for further analysis. DEGs between renal tubulointerstitial tissues of DN patients and normal controls were selected to predict transcription factor genes. The regulatory network between the top 10 transcription factors and their target DEGs was constructed by Cytoscape software. Possible mechanisms on how these TFs might exert their influence on diabetic tubulointerstitial injury via target DEGs were investigated through Gene Ontology (GO) enrichment and Kyoto Encyclopedia of Genes and Genomes (KEGG) pathway analysis. The mRNA expression pattern analysis of transcription factor genes as well as correlation analysis between mRNA expression of transcription factor genes in renal tubulointerstitium and clinical features of DN was performed using Nephroseq v5 online platform. Taken together, a total of 38 transcription factor genes based on 166 DEGs were identified, which may become potential diagnostic biomarkers and therapeutic targets for diabetic tubulointerstitial injury.

## Methods

### Microarray data information

Gene Expression Omnibus (GEO, http://www.ncbi.nlm.nih.gov/geo) is a public genomics data repository storing abundant high throughput gene expression data [16]. The series of GSE30122 [17] was downloaded from GEO database which is based on GPL571(Affymetrix Human Genome U133A 2.0 Array) platform. This microarray data includes 24 normal controls and 10 renal tubulointerstitial tissue samples from DN patients.

### Data preprocessing and differential expression analysis

The raw data were preprocessed by log2 transformation and Z-score normalization. The expression level of genes with more than one probe was averaged. Based on GEO database, we found 166 differentially expressed genes (DEGs) related to diabetic tubulointerstitial injury under DN state. DEGs (adjusted P-value < 0.05 and | log FC (fold change) | > 1) between renal tubulointerstitial tissues of DN patients and healthy controls were screened by limma package [18] in R software. Afterwards, volcano plot of DEGs was drawn by gplots package [19] in R software.

### Identification of transcription factor genes and regulatory network construction of top 10 transcription factors

Transcription factor genes (adjusted P-value < 0.05) involved in diabetic tubulointerstitial injury were selected by UCSC_TFBS (http://www.genome.ucsc.edu/) on Database for Annotation, Visualization and Integrated Discovery (DAVID 6.8, http://david.ncifcrf.gov/). The top 10 TFs were selected based on the number of DEGs associated with each TF. The regulatory network between top 10 transcription factors and their target genes was visualized by Cytoscape software [20] (http://www.cytoscape.org, version 3.7.0) based on the data from UCSC_TFBS.

### Gene ontology (GO) and pathway analyses

As an online bioinformatics database, Database for Annotation, Visualization and Integrated Discovery [21, 22] provides comprehensive functional annotation information on multiple genes. GO enrichment analysis covers categories of biological processes (BP), cellular component (CC) and molecular function (MF) [23]. KEGG is a widely used database in conducting pathway analysis [21]. GO enrichment and KEGG (Kyoto Encyclopedia of Genes and Genomes) pathway analyses of targeted genes that regulated by identified transcription factors were performed using DAVID online tools. Gene count > 2 and P < 0.05 were set as the cutoff value.

### Statistical analysis

mRNA expression pattern [17, 24, 25] of transcription factor genes in renal tubulointerstitium in DN patients compared with normal controls was analyzed by Nephroseq v5 online platform (http://v5.nephroseq.org). Also, Pearson correlation analysis between transcription factor genes and glomerular filtration rate (GFR) [17, 25], serum creatine level (SCR) [24, 25], proteinuria [25], body weight [25] and body mass index (BMI) in renal tubulointerstitium in DN patients was performed. Insignificant results are not shown. Data were checked for compliance with the normal distribution by Shapiro–Wilk test and were expressed as the means ± SDs. Comparisons between 2 groups were performed using unpaired the student’s t test. A two-tailed value of P < 0.05 was considered statistically significant.

## Results

### Screening of DEGs involved in diabetic tubulointerstitial injury

**Fig. 1:**
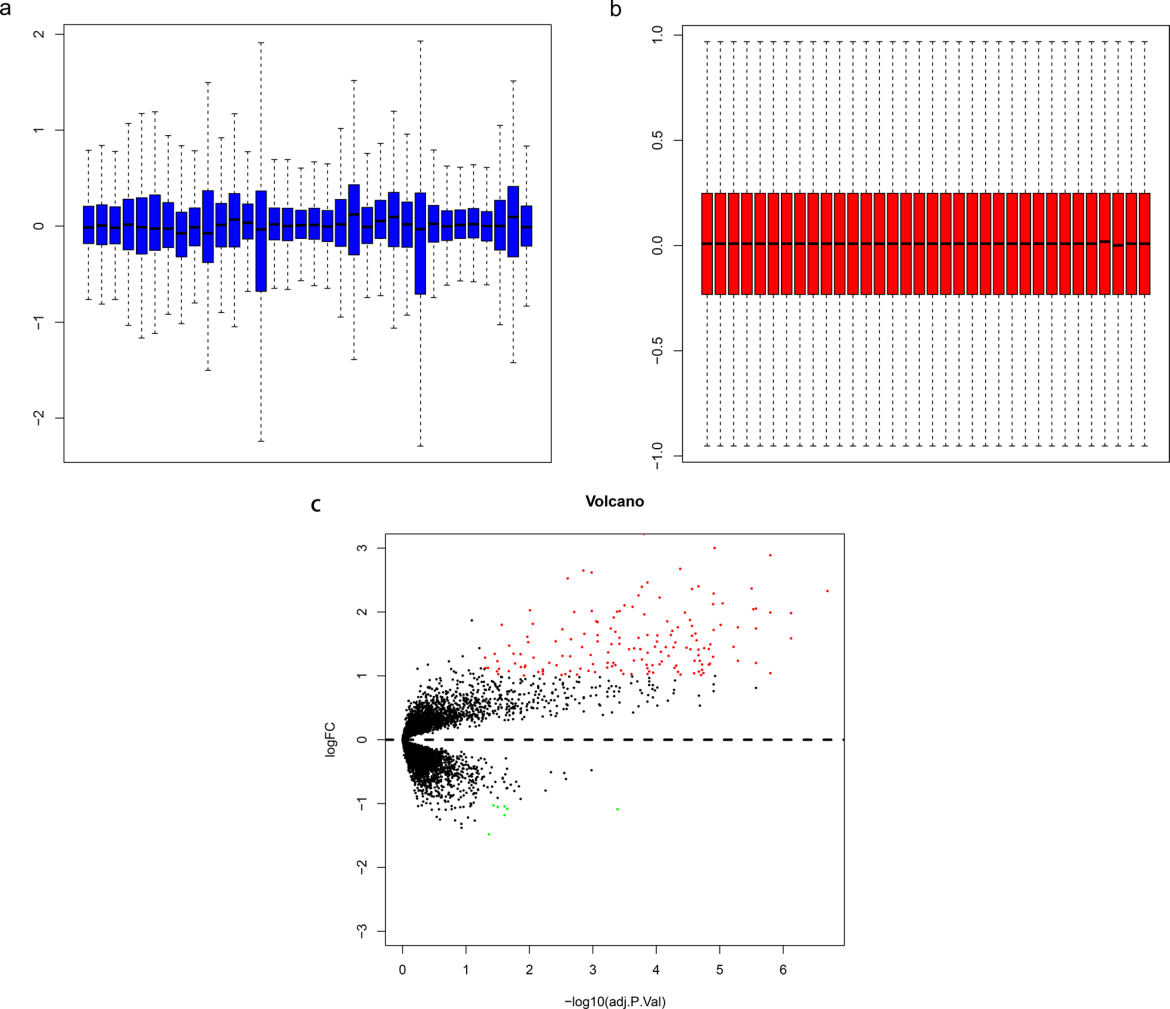
Box plot of normalized data and volcano plot analysis. **a**, **b** Box plot of normalized data from 34 samples. **c** Volcano plot analysis of DEGs. Red dots represent upregulated genes and green dots represent downregulated genes

To identify DEGs related to diabetic tubulointerstitial injury, the mRNA expression microarray (GSE30122) was downloaded from GEO. After normalization of the raw microarray data (Fig. 1a and b), 166 DEGs associated with diabetic tubulointerstitial lesions were identified using limma package as shown in the volcano plot (Fig. 1c). Among them, 159 genes were upregulated and 7 genes were downregulated.

### Identification of transcription factor genes and regulatory network construction of top 10 transcription factors

**Fig. 2:**
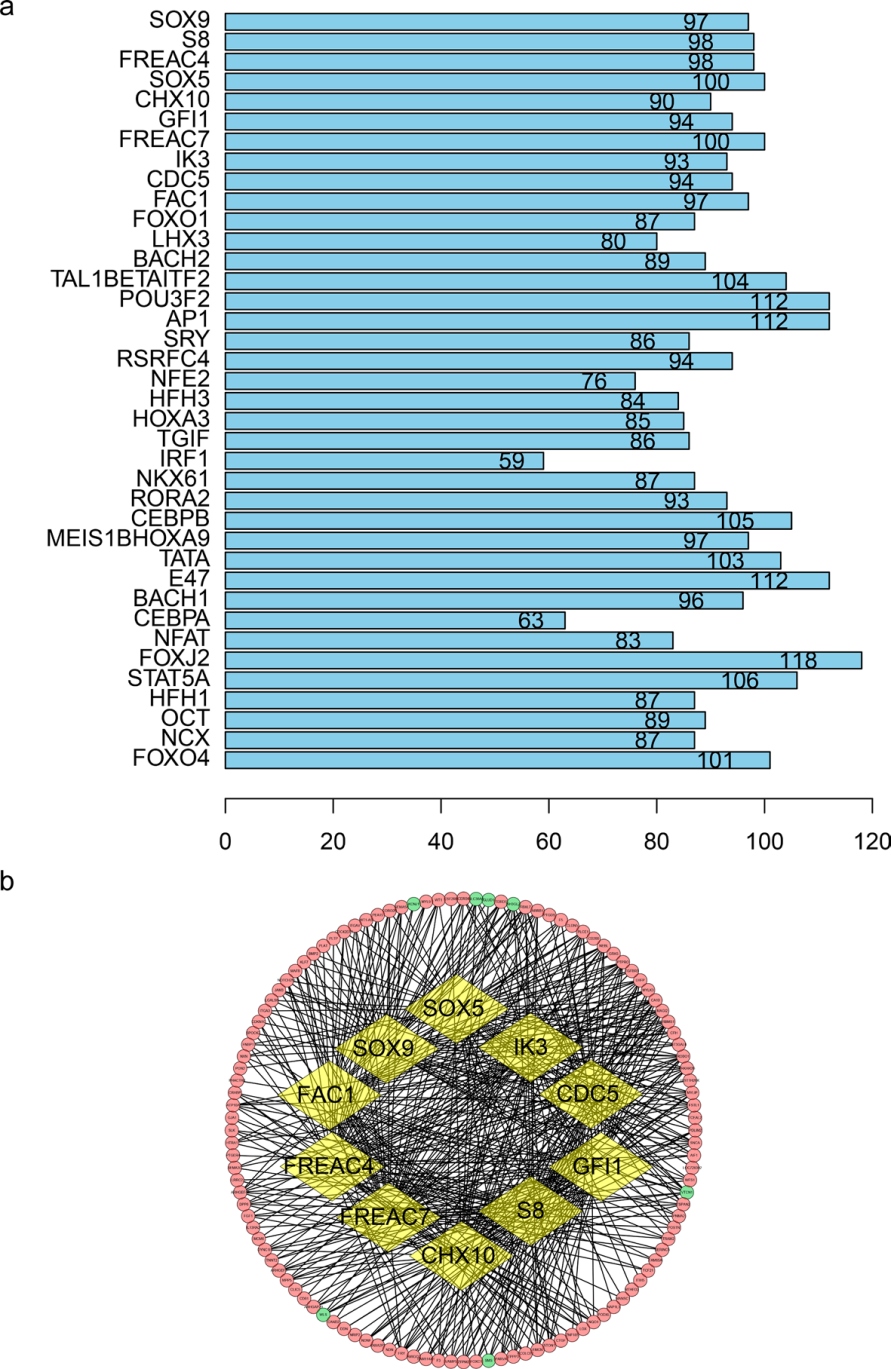
Transcription factor genes identified by UCSC_TFBS and network construction of top 10 transcription factors. **a** Bar plot of 38 transcription factor genes. Numbers in the bar represent the amount of DEGs modulated by corresponding transcription factors. **b** Regulatory network of top 10 transcription factors. Yellow circles represent transcription factors. Red and green circles represent upregulated and downregulated DEGs respectively

To determine transcription factor genes related to diabetic tubulointerstitial injury, UCSC_TFBS online tool on DAVID was employed to identify transcription factor genes that regulate DEGs. As shown in Fig. 2a, a total of 38 transcription factor genes were indicated to be involved in diabetic tubulointerstitial injury. Based on the number of DEGs associated with each TF, the top 10 transcription factors and their target DEGs were applied to create the regulatory network via Cytoscape software. The regulatory network consisted of 500 interactions between 10 transcription factors and 116 DEGs (Fig. 2b).

### GO enrichment analysis of targeted DEGs

**Fig. 3:**
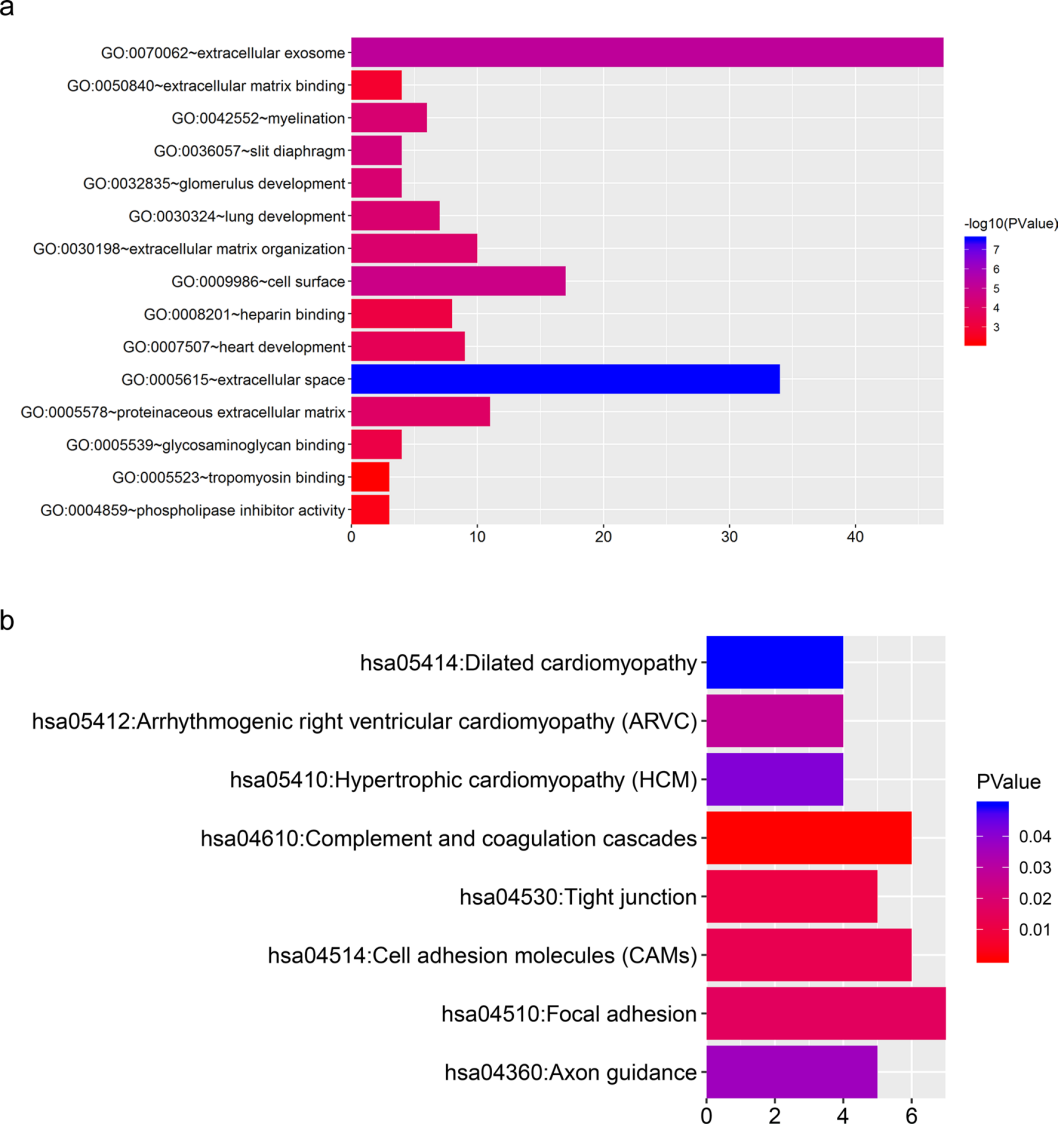
GO enrichment and KEGG pathway analyses of DEGs modulated by identified transcription factors. **a** GO enrichment analysis for targeted DEGs. **b** KEGG pathway analysis for targeted DEGs. GO: Gene Ontology; KEGG: Kyoto Encyclopedia of Genes and Genomes

To investigate biological roles of DEGs modulated by 38 transcription factors, GO enrichment analysis was conducted via DAVID. Myelination (P < 0.001), glomerulus development (P < 0.001), lung development (P < 0.001), extracellular matrix organization (P < 0.001) and heart development (P < 0.001) were the top5 significant enrichment of biological process (Fig. 3a). Extracellular space (P < 0.001), extracellular exosome (P < 0.001), cell surface (P < 0.001), slit diaphragm (P < 0.001) and proteinaceous extracellular matrix (P < 0.001) were the top5 significant enrichment of cell component (Fig. 3a). Glycosaminoglycan binding (P < 0.001), heparin binding (P < 0.001), extracellular matrix binding (P = 0.001), phospholipase inhibitor activity (P = 0.004) and tropomyosin binding (P = 0.006) were the top5 significant enrichment of molecular function (Fig. 3a).

### KEGG pathway analysis of targeted DEGs

To explore the signaling pathways of DEGs modulated by identified transcription factors, KEGG pathway analysis was performed via DAVID. Figure 3b showed that these DEGs were primarily enriched in complement and coagulation cascades (P < 0.001), tight junction (P = 0.010) and cell adhesion molecules (CAMs) (P = 0.012).

### The mRNA expression pattern of transcription factor genes in diabetic renal tubulointerstitium

**Fig. 4:**
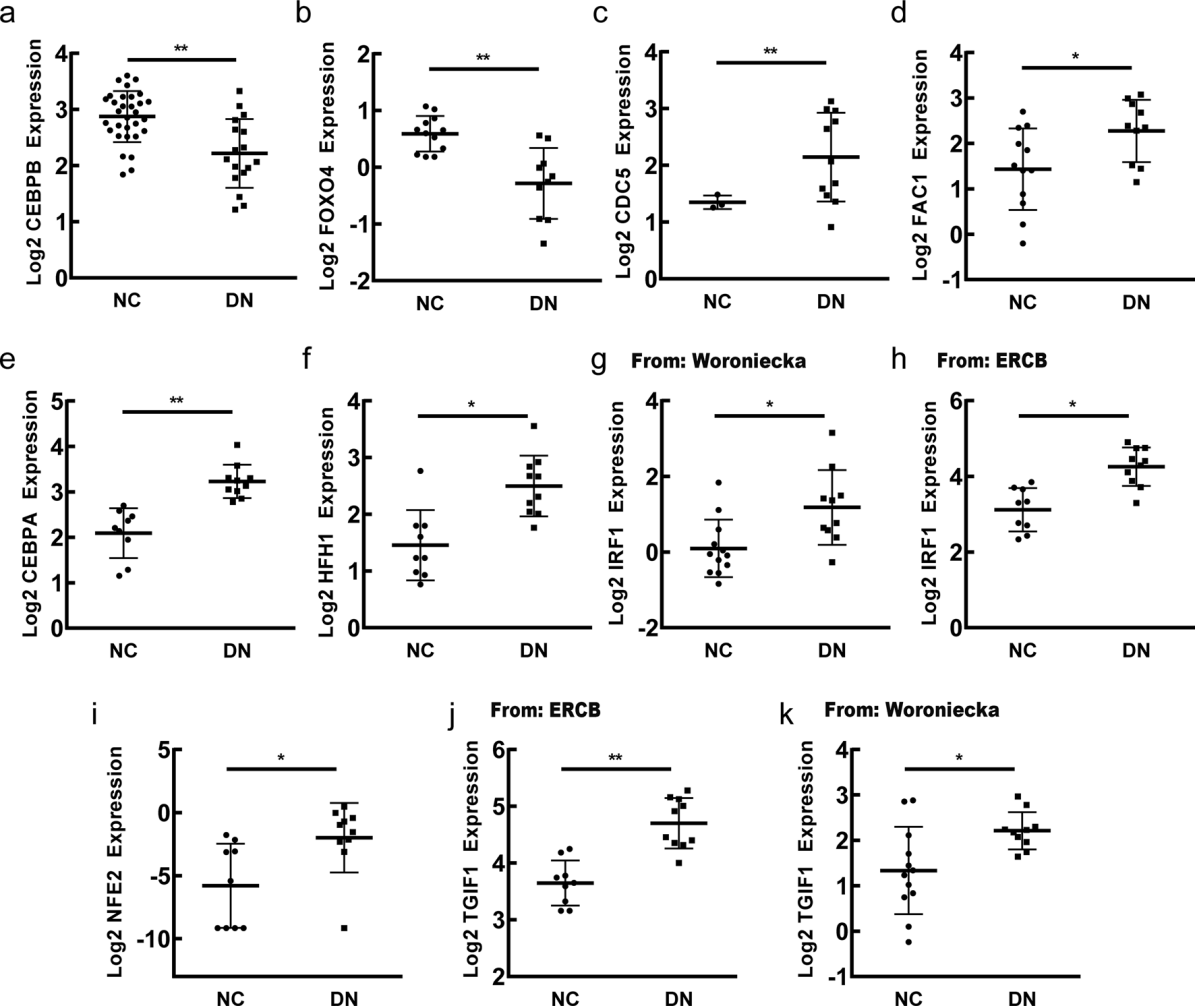
The mRNA expression pattern of transcription factor genes in renal tubulointerstitium of DN patients compared with normal controls. **a**, **b** The decreased mRNA expression of *CEBPB, FOXO4* in DN patients compared with normal controls. **c**–**k** The increased mRNA expression of *CDC5, FAC1, CEBPA, HFH1, IRF1, NFE2, TGIF1* in DN patients compared with normal controls. NC: normal control; DN: diabetic nephropathy. *P < 0.05, **P < 0.01, P < 0.05 was considered statistically significant

To find out the mRNA expression pattern of selected transcription factor genes, relevant analysis was performed by Nephroseq v5 online platform. The results demonstrated that the mRNA expression of *CDC5*, *CEBPA*, *FAC1*, *HFH1*, *IRF1*, *NFE2* and *TGIF1* increased in renal tubulointerstitium of DN patients compared with normal controls while that of *CEBPB* and *FOXO4* decreased in renal tubulointerstitium of DN patients compared with normal controls (Fig. 4).

### Association between mRNA expression of transcription factor genes in renal tubulointerstitium and clinical features of DN

**Fig. 5:**
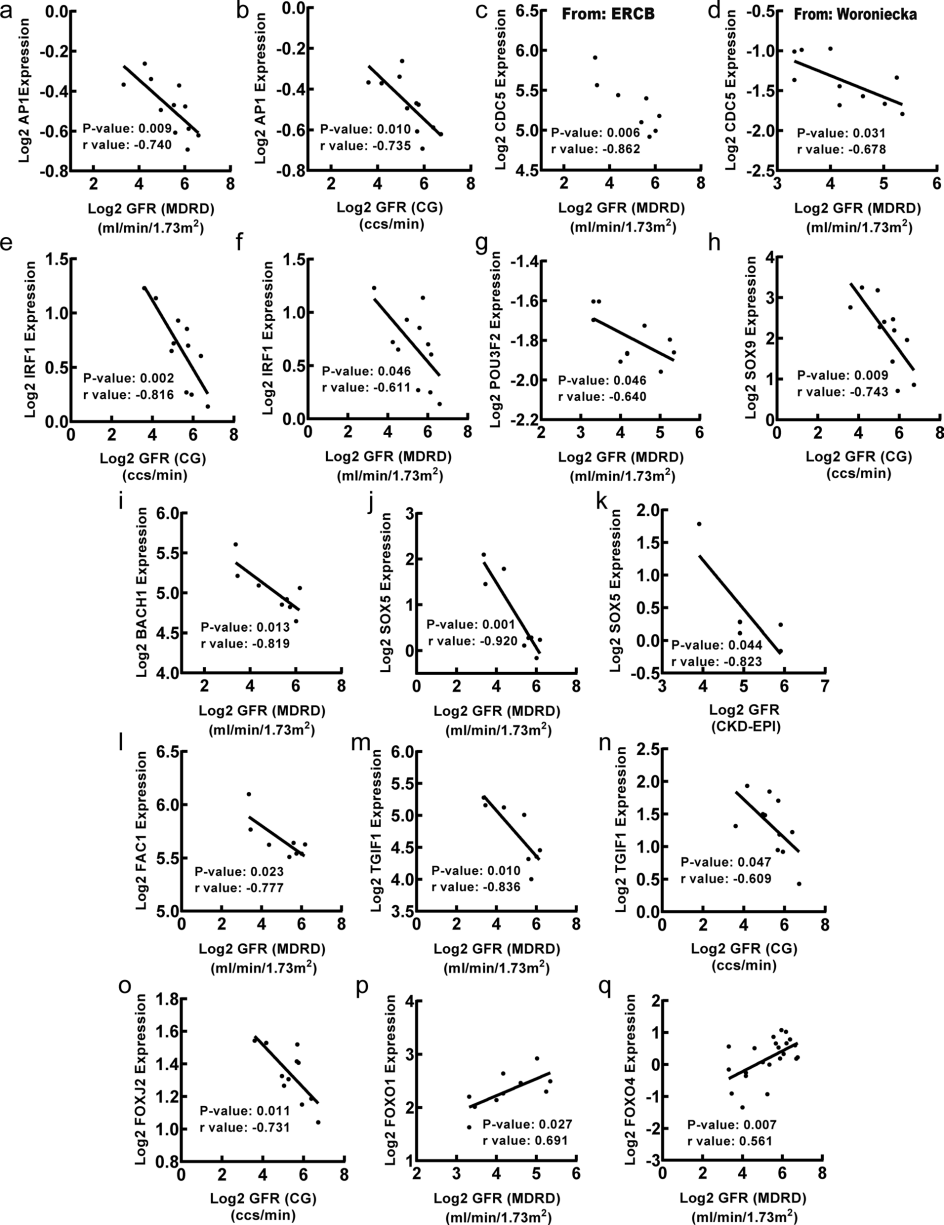
Correlation between mRNA expression of transcription factor genes in renal tubulointerstitium and GFR in DN patients. **a**–**o** The mRNA expression of *AP1 *(p = 0.009, r = − 0.740; p = 0.010, r = − 0.735), *CDC5*(from ERCB: p = 0.006, r = − 0.862; from Woroniecka: p = 0.031, r = − 0.678), *IRF1 *(p = 0.002, r = − 0.816; p = 0.046, r = − 0.611 ), *POU3F2* (p = 0.046, r = − 0.640), *SOX9* (p = 0.009, r = − 0.743), *BACH1* (p = 0.013, r = − 0.819), *SOX5* (p = 0.001, r = − 0.920; p = 0.044, r = − 0.823), *FAC1 *(p = 0.023, r = − 0.777), *TGIF1 *(p = 0.010, r = − 0.836; p = 0.047, r = − 0.609), *FOXJ2 *(p = 0.011, r = − 0.731) negatively correlated with GFR. **p**, **q** The mRNA expression of *FOXO1 *(p = 0.027, r = 0.691), *FOXO4* (p = 0.007, r = 0.561) positively correlated with GFR. p < 0.05 was considered statistically significant. GFR: glomerular filtration rate; MDRD: modification of diet in renal disease; CG: Cockcroft Gault

**Fig. 6:**
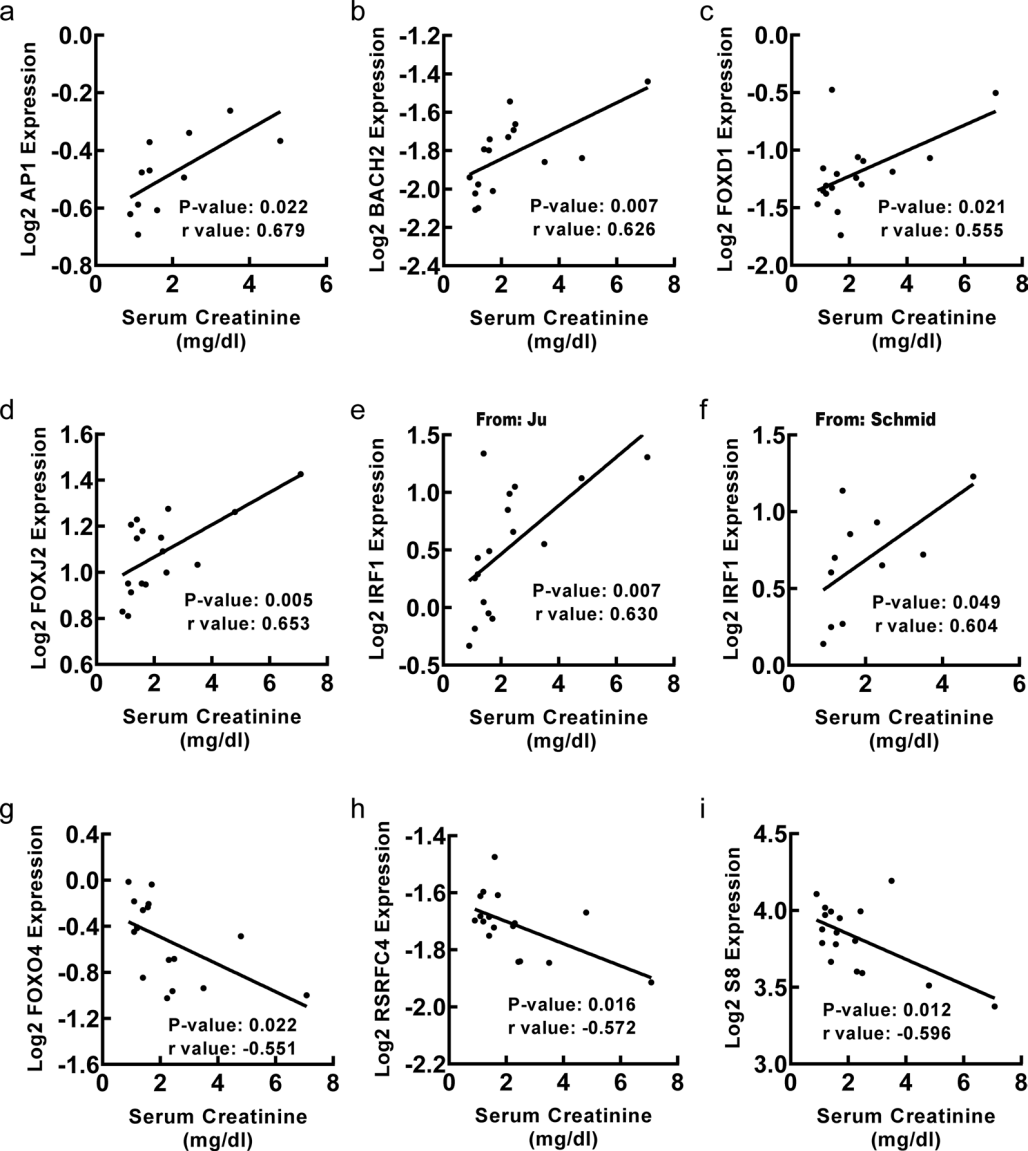
Correlation between mRNA expression of transcription factor genes in renal tubulointerstitium and SCR in DN patients. **a**–**f** The mRNA expression of *AP1 *(p = 0.022, r = 0.679), *BACH2 *(p = 0.007, r = 0.626), *FOXD1 *(p = 0.021, r = 0.555), *FOXJ2 *(p = 0.005, r = 0.653), *IRF1* (from Ju: p = 0.007, r = 0.630; from Schmid: p = 0.049, r = 0.604) positively correlated with SCR. **g**–**i** The mRNA expression of *FOXO4*(p = 0.022, r = − 0.551), *RSRFC4* (p = 0.016, r = − 0.572), *S8 *(p = 0.012, r = − 0.596) negatively correlated with SCR. p < 0.05 was considered statistically significant. SCR, serum creatine

**Fig. 7:**
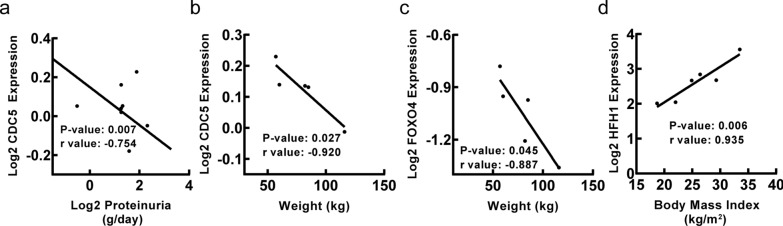
Correlation between mRNA expression of transcription factor genes in renal tubulointerstitium and proteinuria, weight and body mass index in DN patients. **a** The mRNA expression of *CDC5* negatively correlated with proteinuria (p = 0.007, r = − 0.754). **b** The mRNA expression of *CDC5* negatively correlated with weight (p = 0.027, r = − 0.920). **c** The mRNA expression of *FOXO4* negatively correlated with weight (p = 0.045, r = − 0.887). **d** The mRNA expression of *HFH1* positively correlated with body mass index (p = 0.006, r = 0.935). p < 0.05 was considered statistically significant

To explore clinical significance of identified transcription factors in DN, correlation analysis between transcription factor genes and clinical features of DN was conducted by Nephroseq v5 online tool. Firstly, the results showed that mRNA expression of *AP1*, *BACH1*, *CDC5*, *FAC1*, *FOXJ2*, *IRF1*, *POU3F2*, *SOX5*, *SOX9* and *TGIF1* in renal tubulointerstitium reversely correlated with GFR in DN patients (Fig. 5), suggesting that those transcription factor genes may contribute to the progression of DN. Meanwhile, the mRNA expression of *FOXO1* and *FOXO4* in renal tubulointerstitium positively correlated with GFR in DN patients (Fig. 5), indicating that the two transcription factor genes may play a renoprotective role in DN. In this study, MDRD, CG or CKD-EPI GFR were used for further correlation analysis of TFs with GFR as they were used to calculate GFR based on different populations and calculation formulas. The primary data was obtained from different studies, which used different GFR calculation formulas including MDRD, CG and CKD-EPI. Secondly, the mRNA expression of *AP1*, *BACH2*, *FOXD1*, *FOXJ2* and *IRF1* in renal tubulointerstitium positively correlated with SCR in DN patients (Fig. 6), suggesting that those transcription factor genes may promote the progression of DN. Besides, the mRNA expression of *FOXO4*, *RSRFC4* and *S8* in renal tubulointerstitium negatively correlated with SCR in DN patients (Fig. 6), indicating that the three transcription factor genes may have renoprotective roles in DN. Thirdly, the mRNA expression of *CDC5* in renal tubulointerstitium negatively correlated with proteinuria in DN patients (Fig. 7a). Besides, the mRNA expression of *CDC5* and *FOXO4* in renal tubulointerstitium negatively correlated with weight of DN patients (Fig. 7b, c). Moreover, the mRNA expression of *HFH1* in renal tubulointerstitium positively correlated with body mass index in DN patients (Fig. 7d).

## Discussion

Diabetic nephropathy is a globally leading cause of chronic renal failure. In recent years, diabetic tubulopathy has been recognized to have crucial roles in the development of DN [3, 26]. Transcription factors regulate—turn on and off—genes via binding to specific DNA sequences, which are vital for various pathophysiological processes [27]. Such TFs also play key roles in diabetic tubulointerstitial injury as TGF-β [28], HIF-1 [29] and MRTF-A [11]. Although vigorous efforts have been made, the underlying mechanisms of diabetic tubulointerstitial injury still await clarification. The widespread use of microarray technology and bioinformatic methods enables us to identify key transcription factor genes involved in diabetic tubulointerstitial injury, which might yield additional interventional strategies for DN.

A total of 38 transcription factor genes based on 166 DEGs between renal tubulointerstitial tissues of DN patients and normal controls were predicted via UCSC_TFBS. Certain genes such as CEBP and NFAT were observed significantly changed, which corresponded with our earlier researches [10, 30]. As reported, downregulated CCAAT/enhancer binding protein β (C/EBP-β) in db/db mice was proven to induce activated SOCS3/STAT3 signaling pathway, and consequently promote diabetic tubulointerstitial inflammation [30]. Nuclear factor of activated T cell 1 (NFATc1) accompanied with TRPC6 formed a feedback loop to participate in diabetic tubulointerstitial inflammation [10]. Given these validated TFs, it is suggestive to further identify the core TFs and their dominant mechanism, providing reliable research interests. Unfortunately, several indexes including NLRP3, RIPK3, MCP1, KIM-1 and NGAL, were not shown in our analysis, though they were demonstrated might be used as biomarkers of tubular injury in DN condition [31, 32]. Here, we mainly selected DEGs or TFs whose expression change were greater than 2-fold according to the database. It would be worthy to analyze the correlation between these important genes and tubular injury under hyperglycemia condition with more databases.

The regulatory network showed connections between the top 10 transcription factors and their target DEGs. GO enrichment analysis of targeted DEGs demonstrated that extracellular space, extracellular exosome and cell surface were most significantly enriched. The extracellular space refers to the part of a multicellular organism outside the cells, in which extracellular matrix presents. Diabetic tubulointerstitial fibrosis is characterized by increasing deposition of extracellular matrix in the extracellular space [33]. Besides, particular molecules derived from extracellular exosomes have been suggested to serve as potential diagnostic biomarkers in DN including AQP2 [34], AQP5 [34] and let-7c-5p [35]. The loss of molecular binding events between cell surfaces is also involved in diabetic tubulointerstitial fibrosis [36]. Also, GO enrichment analysis displayed that glycosaminoglycan binding and phospholipase inhibitor activity was both significantly enriched, indicating that mitochondria should inevitably become dysfunctional. Correspondingly, increasing data have suggested that persistent mitochondrial dysfunction has a role in the early stages and progression of renal diseases, including diabetic nephropathy [37–40]. Our previous study has also revealed that NRF2/PINK-mediated mitochondrial quality control exerts important effects in diabetic tubular damage and mitochondria-targeted antioxidant MitoQ ameliorates this tubular injury [41]. KEGG pathway analysis of targeted DEGs showed that these DEGs were primarily mapped to complement and coagulation cascades, tight junction and cell adhesion molecules. Existing findings support that activated complement system and procoagulant events contribute to diabetic tubulointerstitial injury [42–45]. An in vitro study conducted in Madin-Darby canine kidney (MDCK) cell line has demonstrated that exposure to high glucose can result in a significant perturbation of the tight junction associated tubular barrier [46]. Tight junctions (TJs) are responsible for adjusting the paracellular transport of solutes and water. They were found to have structural and functional abnormalities in DN condition, impairing the glomeruli, proximal tubules and podocytes [47, 48]. Moreover, cell adhesion molecules such as VCAM-1 [49] and ICAM-1 [50] have been reported to play an important role in diabetic tubulointerstitial injury. Together, all these publications are consistent with our results.

Among 38 transcription factor genes, *CDC5*, *FAC1*, FOXO4, *HFH1*, *IRF1* and *TGIF1* were not only differentially expressed between renal tubulointerstitial tissues of DN patients and normal controls, but also closely related to clinical features of DN. Thus, these 6 candidates may be key transcription factor genes involved in diabetic tubulointerstitial injury. Forkhead box O4 (FOXO4) is a transcription factor involved in the modulation of hypoxia inducible factor 1 subunit alpha (HIF1A) [51], cell cycle [52] and insulin signaling pathway [53]. It has already been recognized as a key transcriptional regulator in DN [54]. Intriguingly, a previous study demonstrated that the induction of FOXO4 was responsible for podocyte apoptosis mediated by advanced glycation end products [55]. However, the results of our study suggested that FOXO4 may have a renoprotective role in diabetic tubulointerstitial injury, raising the possibility that one transcription factor may exert a distinctive effect on different parts of the kidney.

Yet, there is still limited report on the association between other 5 transcription factor genes (*CDC5*, *FAC1*, *HFH1*, *IRF1* and *TGIF1*) and diabetic nephropathy. Cell division cycle 5 like (CDC5) is a DNA-binding protein that regulates cell cycle [56]. *FAC1*, also named as bromodomain PHD finger transcription factor (*BPTF*), is a transcription factor gene related to chromatin remodeling [57]. HFH1 (Forkhead box Q1, FOXQ1) has been reported to mediate epithelial-mesenchymal transition in various human cancers [58]. Interferon regulatory factor 1 (IRF1) is a transcription factor regulating multiple cellular processes, especially for the modulation of interferon (IFN) and IFN-inducible genes [59]. TGFB induced factor homeobox 1 (TGIF1) can act as a transcriptional corepressor of SMAD2 [60] and suppress the function of retinoid X (RXR) receptor [61]. Notably, recent studies have discovered increased TGIF1 can promote the activation of TGF-β1/Smad2/3 signaling pathway, thus contributing to diabetic inflammation and fibrosis in the kidney [62], as well as chronic renal fibrosis [63].

Taken together, translation factors work in many ways to have an impact on diabetic tubulointerstitial injury, despite the mechanisms underlying its role need further exploration. Some limitations in this study should be noted. There are not enough samples from patients and not enough data has not been collected at present. In addition, all the above predicted results should be confirmed by laboratory data. More data and further analysis are encouraged to elucidate those meaningful translational factors and their target genes involved in the inflammatory and fibrotic pathogenesis of DN. With the application of advanced nanomaterials, kidney-targeted therapeutic approaches have rapidly developed [64]. Combining studies on those key molecules and their underlying mechanisms based on bioinformatics analysis, this novelty treatment that precisely intervenes on the target becomes promising.

## Conclusions

In conclusion, this study was intended to search for key transcription factor genes related to diabetic tubulointerstitial injury. A total of 38 transcription factor genes based on 166 DEGs were screened by UCSC_TFBS, which may provide new insights into pathogenesis and potential druggable targets for DN. Of them, *CDC5*, *FAC1*, FOXO4, *HFH1*, *IRF1* and *TGIF1* may be key transcription factor genes. Further experimental studies are needed to confirm our results and delineate biofunctions of those TFs related to diabetic tubulointerstitial injury.

## Supplementary Information

**Additional file 1.** R language for DEGs.

## Data Availability

All data used to support the findings of this study are included within the article. The datasets used and analyzed during the current study are available from GEO GSE30122 (http://www.ncbi.nlm.nih.gov/geo). Source code is available at the “Supplementary Information” section (Additional file 1).

## Abbreviations

DN: Diabetic nephropathy
ESRD: End stage renal disease
TFs: Transcription factors
GEO: Gene Expression Omnibus
DEGs: Differentially expressed genes
GO: Gene Ontology
KEGG: Kyoto Encyclopedia of Genes and Genomes
UCSC_TFBS: A category provided by DAVID
DAVID: The Database for Annotation, Visualization and Integrated Discovery
GFR: Glomerular filtration rate
SCR: Serum creatine level
